# Comparison of Optic Nerve Head Blood Flow Autoregulation among Quadrants Induced by Decreased Ocular Perfusion Pressure during Vitrectomy

**DOI:** 10.1155/2017/6041590

**Published:** 2017-12-07

**Authors:** Ryuya Hashimoto, Tetsuya Sugiyama, Takatoshi Maeno

**Affiliations:** ^1^Department of Ophthalmology, Toho University Sakura Medical Center, 564-1 Shimoshidu, Sakura, Chiba 285-8741, Japan; ^2^Department of Ophthalmology, Osaka Medical College, 2-7 Daigaku-Machi, Takatsuki, Osaka 569-8686, Japan; ^3^Nakano Eye Clinic of Kyoto Medical Co-Operative, 2 Jurakumawari-Higashimachi, Nakagyo-ku, Kyoto 604-8404, Japan

## Abstract

**Purpose:**

The present study aimed to examine changes in optic nerve head (ONH) blood flow autoregulation in 4 quadrants (superior, nasal, inferior, and temporal) with decreased ocular perfusion pressure (OPP) during vitrectomy in order to determine whether there is a significant difference of autoregulatory capacity in response to OPP decrease at each ONH quadrant.

**Methods:**

This study included 24 eyes with an epiretinal membrane or macular hole that underwent vitrectomy at Toho University Sakura Medical Center. Following vitrectomy, the tissue mean blur rate (MBR), which reflects ONH blood flow, was measured. Mean tissue MBRs in the four quadrants were generated automatically in the software analysis report. Measurements were conducted before and 5 and 10 min after intraocular pressure (IOP) elevation of approximately 15 mmHg in the subjects without systemic disorders.

**Results:**

The baseline tissue MBR of the temporal quadrant was significantly lower than that of the other 3 quadrants (all *P* < 0.05). However, the time courses of tissue MBR in response to OPP decrease were not significantly different among the four quadrants during vitrectomy (*P* = 0.23).

**Conclusions:**

There is no significant difference in the autoregulatory capacity of the four ONH quadrants in patients without systemic disorders during vitrectomy.

## 1. Introduction

Autoregulatory mechanisms play very important roles in the control of blood flow in organ tissues to maintain oxygen and nutrition supply through vascular constriction or dilatation [1, 2]. Many studies have reported that autoregulation of optic nerve head (ONH) and choroidal blood flow occurs in response to a change in ocular perfusion pressure (OPP) [3–10].

In general, a role of vascular dysregulation and interaction of OPP and blood flow might be important factors in the eyes with glaucoma [2, 11]. It has been reported that there are circadian intraocular pressure (IOP) variations in healthy or glaucomatous eyes [12]. OPP is the difference between mean blood pressure (MBP) and IOP [2] and several investigators have demonstrated that the progression of glaucoma might be related to fluctuations in OPP [13, 14]. Sung et al. [15] reported that OPP fluctuations during 24 h might be significant predictors for the progression of the central visual field in eyes with normal tension glaucoma. Furthermore, Schmidl et al. [6] reported that impaired autoregulatory capacity was associated with both higher intraocular pressure (IOP) and higher mean blood pressure. However, the mechanism of autoregulation in the ONH blood flow has not been clarified in detail. Considering the above clinical findings, it is important to evaluate this mechanism.

Cull et al. [16] provided evidence for the association between ONH blood flow autoregulation and retinal nerve fiber layer thickness (RNFLT) loss. Another report demonstrated that the number of capillaries was lower in the temporal ONH quadrant than in the other ONH quadrants in monkey eyes with experimental glaucoma [17]. However, no study has elucidated whether there is a significant difference of autoregulatory capacity in the ONH quadrants in response to OPP decrease during vitreous surgery.

The present study aimed to investigate ONH blood flow autoregulation in four quadrants (superior, nasal, inferior, and temporal) in the supine position using laser speckle flowgraphy (LSFG) (LSFG-NAVI-OPE, Softcare Co., Ltd., Fukuoka, Japan) [18–21], during vitrectomy in order to determine whether there is a significant difference of autoregulatory capacity in response to OPP decrease in each ONH quadrant.

## 2. Patients and Methods

### 2.1. Subjects

The present study was approved by the Institutional Review Board/Ethics Committee of Toho University Sakura Medical Center (#2015056). All procedures in this study were in full compliance with the guidelines of the Declaration of Helsinki. All patients were native Japanese, who provided informed consent to participate following a thorough explanation of the nature and possible consequences of this study.

A total of 24 eyes of 24 patients (10 male and 14 female patients) with a mean age of 69.4 ± 6.9 years were enrolled between March 2014 and December 2016.

The subjects in the present study comprised a subset of 18 eyes of 18 patients of a previously published manuscript [19]. Seventeen eyes had an idiopathic epiretinal membrane and seven eyes had a macular hole. All subjects underwent vitrectomy for an idiopathic epiretinal membrane or macular hole at Toho University Sakura Medical Center. The following parameters were within normal limits in all patients (data not shown): systolic/diastolic blood pressure (SBP/DBP), triglyceride level, high/low density lipoprotein cholesterol levels, fasting plasma glucose level, hemoglobin A1c, estimated glomerular filtration rate, and hemoglobin level. Subjects were excluded from participation if they had glaucoma, optic neuropathy, uveitis, or high myopia (refractive error worse than −6 D) and systemic disorders.

### 2.2. Study Protocol

The experimental protocol has been described in detail in previous studies [19–21]. We induced mydriasis by administering 1-2 drops of 0.5% tropicamide and 0.5% phenylephrine hydrochloride (Mydrin-P ophthalmic solution; Santen Pharmaceutical Co., Ltd., Osaka, Japan). One hour before the measurement of ONH blood flow, we also administered pentazocine hydrochloride (Sosegon, 15 mg; Maruishi Pharmaceutical Co., Ltd., Osaka, Japan) and hydroxyzine pamoate (Atarax-P, 25 mg; Pfizer Co., Ltd, Tokyo, Japan) via intramuscular injection.

In all patients, we performed microincision vitrectomy using a 25-gauge instrument with a Constellation vented gas-forced infusion and IOP control system (Alcon, Fort Worth, TX) under local anesthesia, after retrobulbar injection of 2% lidocaine hydrochloride (Xylocaine, 2.5 mL; AstraZeneca K.K., Osaka, Japan) and 0.75% ropivacaine hydrochloride (Anapeine, 2.5 mL; AstraZeneca K.K.).

We first performed cataract surgery and created three infusion ports with a closed valve cannula. Following core vitrectomy, we set two steps of different pressures, that is, lower pressure (0 mmHg) and higher pressure (25 mmHg), using the Constellation vented gas-forced infusion and IOP control system. We measured IOP using Tonopen-AVIA (Reichert, Inc., Buffalo, NY), because this approach yields IOP values that are different from those obtained with the infusion and IOP control system. We used these measurements for the calculation of mean IOP values. SBP and DBP were measured in the same way as reported previously [19–21].

ONH blood flow was measured thrice consecutively using LSFG-NAVI-OPE at lower pressure at baseline and 5 and 10 min after IOP elevation. The 5- and 10-min time points for measurement of ONH blood flow were determined using preliminary results obtained from 13 of the 24 studied eyes. In these preliminary experiments, ONH blood flow was measured 2, 5, and 10 min after raising IOP. Because ONH blood flow was lowest at 5 min, we decided to only measure ONH blood flow at 5 and 10 min after IOP elevation.

### 2.3. Measurement and Analysis of ONH Blood Flow

LSFG was developed to facilitate the noncontact analysis of ocular blood flow using the laser speckle phenomenon [22]. The mean blur rate (MBR), which indicates ONH blood flow [23, 24], was obtained using LSFG-NAVI-OPE in the supine position. Recent studies have shown that the MBR in the ONH measured using LSFG was significantly correlated with actual ONH blood flow measured using the microsphere method in monkey eyes [25] and using the hydrogen gas clearance method in rabbits [26, 27]. Furthermore, LSFG is a useful devise to examine the blood flow in the ONH [19–27], retinal vessels [28–30], and choroid [31–34].

**Figure 1:**
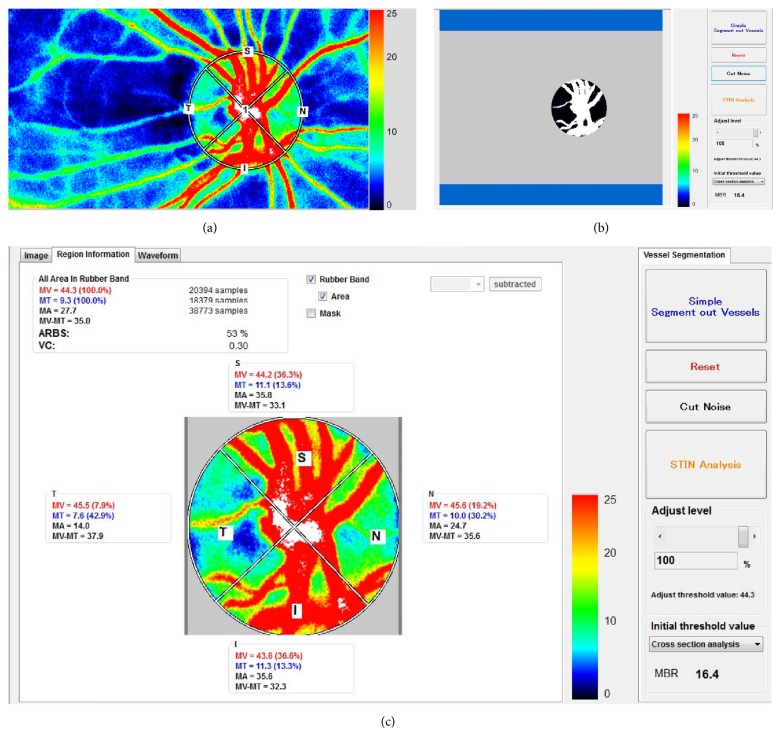
*Analysis of circulation in the optic nerve head by laser speckle flowgraphy analyzer software*. (a) The margin of the optic nerve head (ONH) is identified using a circular or elliptical boundary. (b) LSFG Analyzer software (Ver. 3.1.79.0, Softcare, Ltd.) can distinguish the vessels and tissues using the automated definitive threshold. The white area indicates the vessel area while the black area indicates the tissue area. (c) Blood flow in the ONH quadrants (S: superior, I: inferior, N: nasal, and T: temporal) is calculated by using LSFG Analyzer software. MT means the tissue mean blur rate in the ONH.

First, we identified the margin of the ONH using a circular or elliptical boundary (Figure 1(a)). ONH circulation can be automatically divided into vessels and tissues using the automated definitive threshold via the LSFG Analyzer software (Ver. 3.1.79.0, Softcare, Ltd.) (Figure 1(b)). The MBR of the ONH tissue (tissue MBR) has been shown to be linearly correlated with capillary blood flow, regardless of fundus pigmentation [27]. The MBR was calculated for each quadrant by dividing the tissue MBR in each quadrant (i.e., superior, nasal, inferior, and temporal) by the entire tissue MBR in the ONH (Figure 1(c)). The measurement was performed thrice consecutively at each time point using LSFG-NAVI-OPE, and the mean values of tissue MBR were calculated from these measurements using the LSFG Analyzer software.

### 2.4. Measurement of Intraoperative Intraocular Pressure, Blood Pressure, Pulse Rate, and Oxygen Saturation

The intraoperative IOP, blood pressure, pulse rate, and oxygen saturation (SpO2) were measured simultaneously with ONH blood flow using a method reported previously [19–21]. The mean blood pressure (MBP) was calculated from SBP/DBP according to the following formula [12]: MBP = 1/3 × (SBP − DBP) + DBP. OPP was calculated using the following formula [12]: OPP (supine position) = 115/130 × MBP − IOP.

### 2.5. Statistical Analysis

All data are presented as mean ± standard deviation. One-way repeated-measures analysis of variance (ANOVA) was used to analyze the changes in tissue MBR from the baseline for each ONH quadrant. Differences in OPP were tested for statistical significance using one-way measures ANOVA. Two-way repeated measures ANOVA was used to analyze the time course of tissue MBR for each ONH quadrant. All statistical analyses were performed using the SPSS statistical software (Ver. 23, IBM Corp., Armonk, NY). A *P* value < 0.05 was considered to be statistically significant.

## 3. Results

### 3.1. Time Course of Intraoperative Intraocular Pressure, Ocular Perfusion Pressure, Pulse Rate, and Oxygen Saturation

Table 1 shows the time course of intraoperative IOP, OPP, pulse rate, and SpO2. The IOP significantly increased from baseline to 5 and 10 min as the infusion pressure increased. Accordingly, OPP was significantly lower at 5 min (50.4 ± 11.0 mmHg) and 10 min (49.1 ± 9.8 mmHg) than at baseline (67.0 ± 10.2 mmHg). There were no significant differences in the MBP, pulse rate, and SpO2.

### 3.2. Comparison of Baseline ONH Blood Flow in the Tissue Area

**Figure 2:**
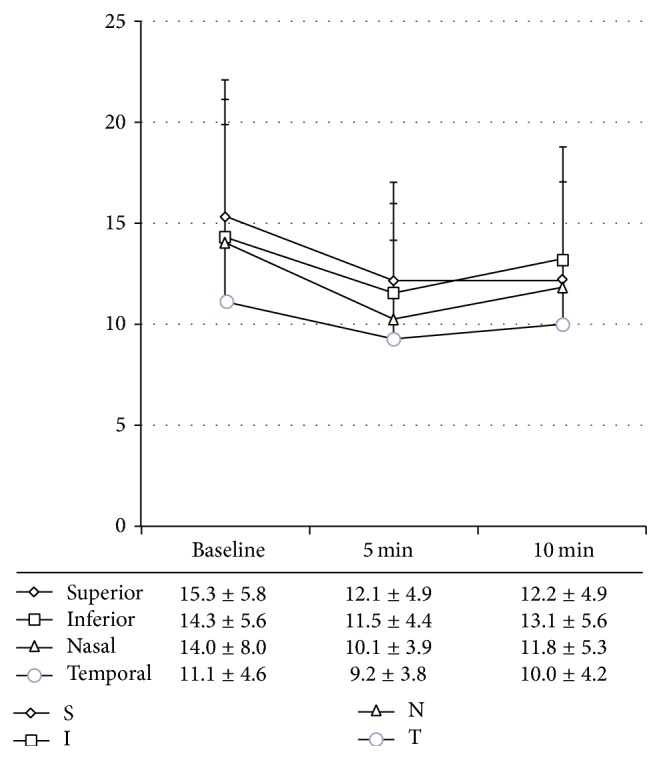
*Time course of tissue MBR changes in the four quadrants*. Data are expressed as mean ± standard deviation for 24 subjects. Results of statistical analysis are described in the text. S: superior, I: inferior, N: nasal, and T: temporal.

We compared the baseline tissue MBR among each ONH quadrant because tissue MBRs were measured in the same individual and section. The baseline tissue MBR of the temporal quadrant was significantly lower than that of the other three quadrants (all *P* < 0.05, one-way repeated measures ANOVA with Bonferroni correction) (Figure 2).

### 3.3. Time Course of ONH Blood Flow Change in Each Quadrant

Figure 2 shows the tissue MBRs in the four quadrants at each measurement point.

Two-way repeated measures ANOVA showed that both the time and quadrant effects were significant (both *P* < 0.01). Regarding the time effect, pairwise comparison analysis showed that there were significant differences between the baseline and the 5-min and 10-min time points after IOP elevation (*P* < 0.01, *P* = 0.01, respectively). Furthermore, there was a significant difference between the 5-min and the 10-min time points after IOP elevation (*P* < 0.01, Bonferroni). Regarding the quadrant effect, pairwise comparison analysis showed that tissue MBR in the temporal quadrant was significantly lower than that in the superior and inferior quadrants (both *P* < 0.01). In contrast, there was no significant difference between the temporal and nasal quadrant, the superior and inferior quadrants, the superior and nasal quadrants, and the inferior and nasal quadrants (*P* = 0.12, 1.00, 0.69, and 0.88, Bonferroni).

Finally, no significant interaction was detected between the time courses of tissue MBR and each quadrant (*P* = 0.23).

## 4. Discussion

The current study showed that no significant interaction was detected between the time courses of tissue MBR and each quadrant. That finding indicated that there was no significant difference of the autoregulatory capacity in each ONH quadrant in response to OPP decrease during vitreous surgery in patients without systemic disorders.

Regarding ONH tissue blood flow in healthy subjects, there have been several investigations concerning autoregulation in response to OPP fluctuations using LSFG [10, 35, 36]. Witkowska et al. [10] recently reported ONH tissue blood flow regulation in response to OPP elevation during isometric exercise using LSFG. In view of that autoregulatory mechanism operated in response to OPP fluctuations, our result is almost consistent with previous studies. However, the blood flow autoregulation in each ONH quadrant tissue area in response to OPP decrease during vitrectomy had not been previously investigated. In general, the short posterior ciliary arteries and the circle of Zinn-Haller, partly via the peripapillary choroid, supply the lamina cribrosa with nutrients [37]. It is considered that tissue MBR obtained by the LSFG analysis software might present the blood flow of microvessels or branches of these arteries in the lamina cribrosa [24]. Although the reason has yet to be clarified, there was no significant difference of autoregulatory capacity of each ONH quadrant tissue blood flow in patients without systemic disorders. Further study with more cases in patients with diseases such as glaucoma will be needed to evaluate the mechanism.

On the other hand, Chan et al. [38] recently reported that systemic disorders, such as cardiovascular diseases, might be a strong risk factor for rapid glaucoma progression. Dysfunction of ONH blood flow autoregulation was thought to be one of the risk factors for the progression of primary open-angle glaucoma as well as elevation of IOP [39]. Furthermore, it is known that glaucomatous damage occurs preferentially in the temporal ONH quadrant [40] and tissue MBR of the temporal, superior, and inferior quadrants has been reported to be strongly associated with the RNFLT in patients with glaucoma [41]. Considering these clinical findings, the autoregulatory mechanism in each ONH quadrant in patients without glaucoma or systemic diseases might be different from that in patients with these diseases.

Our results also demonstrated that the baseline tissue MBR in the temporal quadrant was lower than that of the other quadrants. Furthermore, pairwise comparison analysis showed that the tissue MBR in the temporal quadrant was lower than that in the superior or inferior quadrants. A previous study using a laser Doppler flowmeter reported that ONH blood flow of the temporal quadrant in eyes without glaucoma was significantly lower than that of the nasal quadrant [42]. In another study on animal eyes with experimental glaucoma, it was reported that there was a reduction in the number of capillaries in the temporal ONH quadrants [17]. Our result is consistent with the finding of these previous reports. On the other hand, the RNFLT of the temporal quadrant has been reported to be thinner than that of the other quadrants in normal subjects [43, 44]. Lower ONH blood flow in the temporal quadrant might be related with a thinner RNFLT in the same quadrant.

The present study had some limitations. First, we did not clarify whether a macular hole or epiretinal membrane influenced the autoregulatory mechanism in the ONH because all subjects had these conditions in the present study. Second, all subjects were given sedatives an hour before measurement. Further studies are needed to elucidate whether administration of sedatives can influence the autoregulatory capacity in each ONH quadrant during vitrectomy. Third, further investigations will be needed to evaluate whether we can use the value of MBR as an absolute value, although tissue MBR of the ONH has been shown to be highly correlated with absolute blood flow values, measured with the microsphere method or the hydrogen gas clearance method. Finally, our results were obtained using a relatively small number of subjects. Further studies with more subjects are required to determine which ONH quadrant is more susceptible to a decrease in OPP during vitrectomy.

## 5. Conclusions

In summary, there is no significant difference of autoregulatory capacity in the ONH quadrants in response to OPP decrease during vitrectomy in subjects without systemic disorders.

## Figures and Tables

**Table 1:** Intraoperative intraocular pressure, intraocular pressure, mean blood pressure, ocular perfusion pressure, pulse rate, and oxygen saturation.

	Baseline	5 min	10 min
IOP (mmHg)	14.8 ± 2.5	29.4 ± 3.8^*∗*^	29.1 ± 2.8^*∗*^
MBP (mmHg)	92.4 ± 10.5	90.3 ± 10.8	88.4 ± 10.0
OPP (mmHg)	67.0 ± 10.2	50.4 ± 11.0^*∗*^	49.1 ± 9.8^*∗*^
Pulse rate (beats/min)	65.8 ± 10.3	66.2 ± 9.9	65.2 ± 10.1
SpO_2_ (%)	94.9 ± 1.9	94.8 ± 2.3	94.7 ± 2.2

Data are expressed as mean ± standard deviation for the 24 subjects; IOP, intraocular pressure; MBP, mean blood pressure; OPP, ocular perfusion pressure; SpO_2_, oxygen saturation; ^*∗*^significant difference from the baseline level (corrected *P* < 0.05 following a Bonferroni test).
